# The Golgi Apparatus and its Next-Door Neighbors

**DOI:** 10.3389/fcell.2022.884360

**Published:** 2022-04-28

**Authors:** Akihiko Nakano

**Affiliations:** RIKEN Center for Advanced Photonics, Wako, Japan

**Keywords:** Golgi, membrane traffic, ERGIC, GECCO, trans-Golgi network, recycling endosome, live imaging, SCLIM

## Abstract

The Golgi apparatus represents a central compartment of membrane traffic. Its apparent architecture, however, differs considerably among species, from unstacked and scattered cisternae in the budding yeast *Saccharomyces cerevisiae* to beautiful ministacks in plants and further to gigantic ribbon structures typically seen in mammals. Considering the well-conserved functions of the Golgi, its fundamental structure must have been optimized despite seemingly different architectures. In addition to the core layers of cisternae, the Golgi is usually accompanied by next-door compartments on its *cis* and *trans* sides. The *trans*-Golgi network (TGN) can be now considered as a compartment independent from the Golgi stack. On the *cis* side, the intermediate compartment between the ER and the Golgi (ERGIC) has been known in mammalian cells, and its functional equivalent is now suggested for yeast and plant cells. High-resolution live imaging is extremely powerful for elucidating the dynamics of these compartments and has revealed amazing similarities in their behaviors, indicating common mechanisms conserved along the long course of evolution. From these new findings, I would like to propose reconsideration of compartments and suggest a new concept to describe their roles comprehensively around the Golgi and in the post-Golgi trafficking.

## Introduction

The Golgi apparatus has been recognized as a central station of intracellular membrane traffic because it stands at the intersection of secretory, lysosomal/vacuolar, and endosomal transport routes (Jamieson and Palade, 1966; Farquhar and Palade, 1998). Its beautiful structure comprising a layer of cisternae has attracted many cell biologists. However, the more we know about its functions, the more questions confront us leading to very active debates.

**FIGURE 1:**
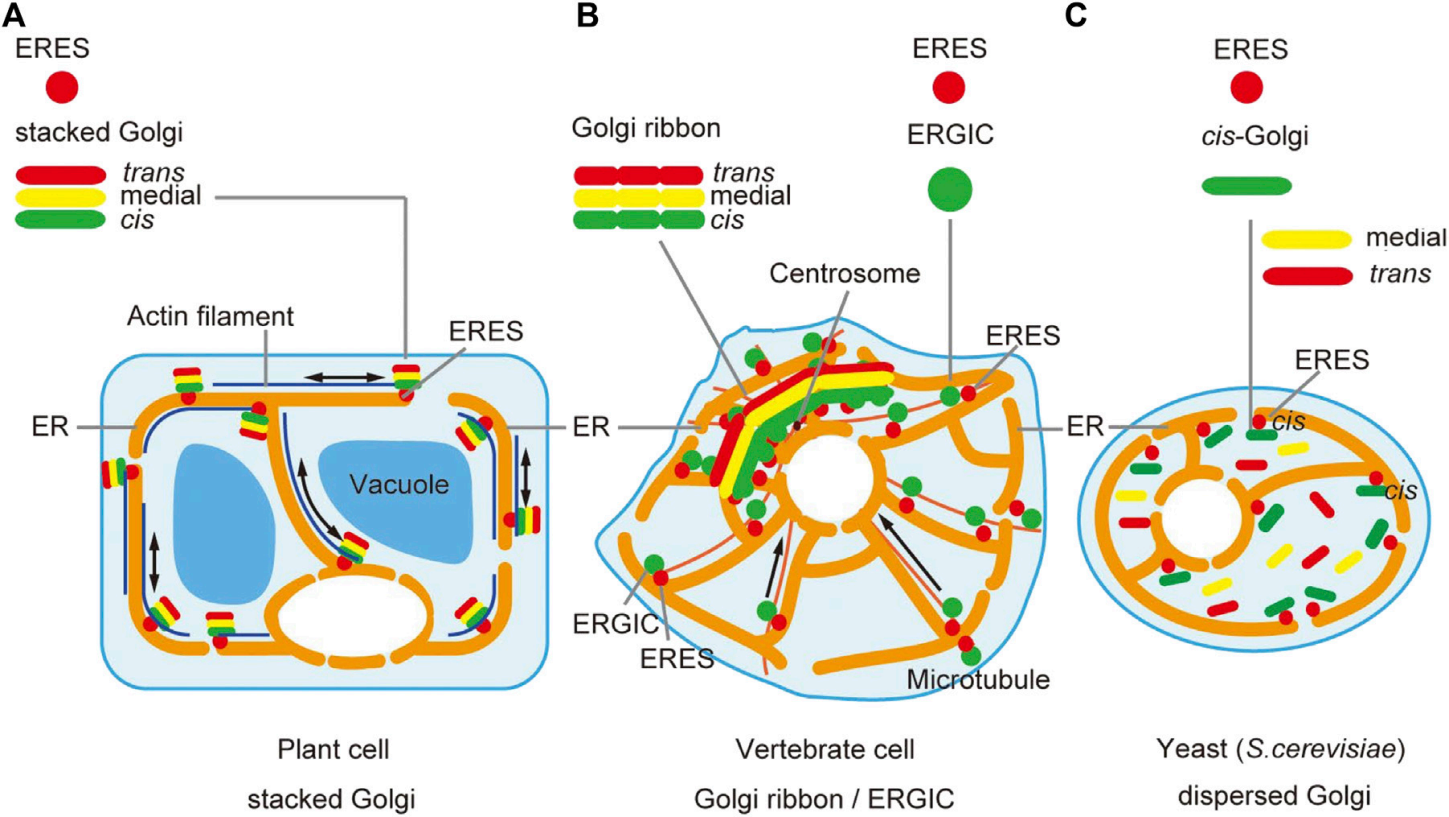
Spatial organization of the Golgi. The spatial arrangement of the Golgi apparatus looks different among species. **(A)** In plant cells, Golgi cisternae (*cis*, medial, and *trans*) are beautifully layered in single stack units, which are scattered in the cytoplasm. Model animals of invertebrates, Drosophila and *C. elegans*, show a similar pattern. **(B)** In vertebrate animals like mammals, Golgi stacks are concentrated at the perinuclear centrosomal region often in an interconnected fashion to form a gigantic Golgi ribbon structure. **(C)** In the budding yeast *S. cerevisiae*, Golgi cisternae do not stack and are individually scattered in the cytoplasm. Note that ERES (ER exit sites) are very frequently observed in the very vicinity of the Golgi stack **(A)** or ERGIC (ER-Golgi intermediate compartment) **(B)** or *cis*-Golgi **(C)** [modified from Kurokawa et al. (2019)].

As will be discussed below, the organization of the Golgi differs considerably among different species, making it difficult to draw a comprehensive picture of this organelle (Figure 1). A simple model shown in many textbooks describes a stack of several flattened cisternae with a polarity from *cis* to *trans*, but such a typical structure is not always seen in living cells.

The best-known Golgi function is its role as a glycosylation factory. Many secretory proteins have *N*- and/or *O*-linked oligosaccharide chains, which are subjected to complicated modification reactions (Berger and Rohrer, 2008; Stanley, 2011). In plants, the synthesis of cell wall polysaccharides is also an important task of the Golgi (Driouich et al., 1993; Hoffmann et al., 2021). To fulfill these biochemical reactions, the array of Golgi cisternae is differentiated into sub-compartments and zones to achieve the best performance (Dunphy and Rothman, 1985; Tie et al., 2018; Tojima et al., 2019).

In addition to the important function of glycosylation, the Golgi is known to act as a sorting platform. It receives a variety of proteins from the ER and recycles some of them back to the ER (Nilsson et al., 1989; Pelham and Munro, 1993; Letourneur et al., 1994; Sato et al., 1995, 1997; Sato et al., 1996). There are a variety of destinations for cargo transiting the Golgi, involving many different carriers to the plasma membrane and other organelles (Griffiths and Simons, 1986; Weiss and Nilsson, 2000; Rodriguez-Boulan and Müsch, 2005; Bonifacino, 2014; Dell’Angelica and Bonifacino, 2019). Recent studies have indicated that these sorting functions should be mostly ascribed to specialized regions proximal to and distal to the main body of the Golgi. Names given to these compartments are IC (intermediate compartment) or ERGIC (ER-Golgi intermediate compartment) on the *cis*-side (Schweizer et al., 1990; Saraste and Svensson, 1991; Plutner et al., 1992; Klumperman et al., 1998) and TGN (*trans*-Golgi network) on the *trans*-side (Roth et al., 1985; Griffiths and Simons, 1986; Taatjes and Roth, 1986; Geuze and Morré, 1991). The purpose of this review is to provide insights into the common features of these neighboring compartments, from a comparative view of yeast, plant, and animal cells.

One famous and memorable debate regarding trafficking in the Golgi dealt with the question of how cargo molecules are conveyed within the Golgi (Mellman and Simons, 1992; Glick and Malhotra, 1998; Pelham and Rothman, 2000). Live imaging of the budding yeast provided strong support for the cisternal maturation model (Losev et al., 2006; Matsuura-Tokita et al., 2006; Glick and Nakano, 2009; Nakano and Luini, 2009; Glick and Luini, 2011), but many unanswered questions remain. A group of researchers working on such critical Golgi issues gathered in Barcelona in 2009, mutually exchanging ideas and proposing future directions. The summary of the meeting review (Emr et al., 2009) concluded that “advances in high-resolution microscopy and biochemical techniques will help to clarify this issue.” As one of those who were present, and one who has seriously pursued the development of high-speed and super-resolution live imaging microscopy, I would like to summarize what we know now and propose some concepts emerging from the new knowledge. The Golgi and its next-door neighbors, as well as selected aspects of post-Golgi trafficking will be discussed in this review article. For the details of our imaging technology (SCLIM; super-resolution confocal live imaging microscopy) (see Footnote for abbreviations), I refer to our reviews published elsewhere (Nakano, 2002; Kurokawa et al., 2013; Kurokawa and Nakano, 2020; Tojima et al., 2022).

### Spatial Organization of the Golgi

Many studies on membrane trafficking have been performed on mammalian cells, whose Golgi typically forms a giant structure called the Golgi ribbon in the perinuclear area near the centrosome (Ladinsky et al., 1999; Thyberg and Moskalewski, 1999; Rios and Bornens, 2003) (Figure 1B). Formation of Golgi ribbon depends on dynein-motor-driven gathering of membranes along microtubules and thus depolymerization of microtubules causes the scattering of hundreds of “Golgi ministacks” in the cytoplasm. This kind of organization has been thought specific to vertebrates, which have evolved radial patterning of microtubules. Indeed, invertebrate model animals, such as Drosophila and *C. elegans,* have scattered ministack-type of Golgi. Concentration of the Golgi near the centrosome is considered advantageous for massive protein secretion in a polarized fashion, for example, during cell migration, but why it forms the ribbon structure remains elusive (Terasaki, 2000; Marra et al., 2007; Yadav et al., 2009; Wei and Seemann, 2010; Yadav et al., 2011; Ferraro et al., 2014; Saraste and Prydz, 2019). The gigantic Golgi ribbon structure makes it sometimes difficult to apply detailed live imaging.

Plant cells, in contrast, provide ideal systems to study the Golgi at high resolution (Ito et al., 2014) (Figure 1A). Beautiful Golgi stacks are separately seen in the cytoplasm in their natural state and EM studies reveal details of their structure (Zhang and Staehelin, 1992; Staehelin and Kang, 2008; Donohoe et al., 2013; Robinson, 2020). Furthermore, we have relied on this advantage of plant cells to apply live imaging of Golgi and its neighboring compartments, as will be discussed in detail in this review.

The budding yeast *S. cerevisiae* is another extreme example (Figure 1C). The Golgi in this organism does not form stacks but exists as individual cisternae scattered in the cytoplasm (Preuss et al., 1992; Rambourg et al., 2001; Beznoussenko et al., 2016). This feature gave a great advantage for the demonstration of cisternal maturation by live imaging (Losev et al., 2006; Matsuura-Tokita et al., 2006). It appears that *S. cerevisiae* has somehow abandoned stacking of Golgi cisternae, because there exist species of budding yeast that have stacked Golgi (Rambourg et al., 1995; Glick, 1996; Rossanese et al., 1999). Nevertheless, *S. cerevisiae* has a very efficient secretory activity and why it copes without stacking remains a big mystery. Although the mechanisms and meanings of Golgi cisternal stacking are not amenable to study in this organism, it is still a fascinating choice to work with because of the invaluable molecular knowledge accumulated on trafficking machinery and the availability of various techniques including powerful genetics (Rothblatt and Schekman, 1989; Schekman, 1992; Nakano, 2008; Jackson, 2009; Papanikou and Glick 2009; Suda and Nakano, 2012).

The comparison of typical spatial arrangement of the Golgi in plant, animal, and yeast cells is summarized in Figure 1, giving an impression of how much they are different. However, despite the architectural differences, the important message of this review emphasizes how conserved the trafficking mechanisms are among them.

### ER-Golgi Interface

Let’s think about the cargo delivery process from the ER to the Golgi. It is well known that the assembly of the coat protein complex II (COPII) plays an essential role in the formation of the vesicles (COPII vesicles) that export cargo proteins from the ER (Barlowe et al., 1994; Sato and Nakano, 2007). Activation of Sar1 GTPase by its guanine-nucleotide exchange factor (GEF) Sec12 is thought to be the most upstream reaction in the vesicular journey of the secretory pathway (Nakano et al., 1988; Nakano and Muramatsu, 1989; Barlowe and Schekman, 1993). The specialized places on the ER forming COPII vesicles are called transitional ER (tER) or ER exit sites (ERES) (Palade, 1975; Hughes and Stephens, 2008; Budnik and Stephens, 2009; Okamoto et al., 2012; Kurokawa and Nakano, 2019). In a simple scenario, the COPII vesicles released from the ERES into the cytoplasm somehow find and reach the Golgi to hand over cargo. However, this is not easy in a large mammalian cell, because a large number of ERES are present at the cell periphery and the Golgi is concentrated near the centrosome. There is a very long way to go.

The intermediate compartment between the ER and Golgi (IC/ERGIC) was first defined as a compartment where a unique marker protein (rat p58 or human ERGIC53) is present (Schweizer et al., 1990; Saraste and Svensson, 1991; Plutner et al., 1992; Klumperman et al., 1998; Hauri et al., 2000; Ying et al., 2000; Ben-Tekaya et al., 2005; Appenzeller-Herzog and Hauri, 2006). A complex structure containing both COPII and COPI (coat protein complex I) was discovered by immuno-EM studies and named vesicular tubular cluster (VTC) and proposed to be the place where cargo sorting takes place between the ER and the Golgi (Bannykh et al., 1996; Martínez-Menàrguez et al., 1999). While ERGIC appeared to be a stable compartment and VTC was first regarded as a transient structure, they are most likely referring to the same intermediate compartment connecting the traffic from the ERES to the *cis* face of the Golgi. Such an intermediate compartment was not defined in yeast and plant cells until recently. It should be noted here that ERGIC is frequently observed in the vicinity of ERES (Sannerud et al., 2006; Budnik and Stephens, 2009; Saraste and Marie, 2018).

Experiments using brefeldin A (BFA), an inhibitor of GEF toward Arf GTPases, give us hints on the commonness of trafficking mechanisms (Takatsuki and Tamura, 1985; Klausner et al., 1992). Arf GTPases are relatives of Sar1 and are required for formation of the vesicles coated with COPI, adapter protein complexes (AP-1∼5), and GGAs (Golgi-localized, γ-ear-containing, Arf-binding family of proteins) (Jackson and Casanova, 2000; Pucadyil and Schmid, 2009; Dacks and Robinson, 2017; Dell’Angelica and Bonifacino, 2019). COPI vesicles are involved in retrograde traffic in the Golgi and in the Golgi-to-ER recycling (Letourneur et al., 1994; Rabouille and Klumperman, 2005; Donohoe et al., 2007). Treatment with BFA causes blockade of COPI vesicle formation and induces enormous reorganization of endomembrane systems. Remarkable studies on mammalian cells by Lippincott-Schwartz et al. (1990), Lippincott-Schwartz et al. (1991) demonstrated that the Golgi was almost completely absorbed into the ER upon BFA treatment.

**FIGURE 2:**
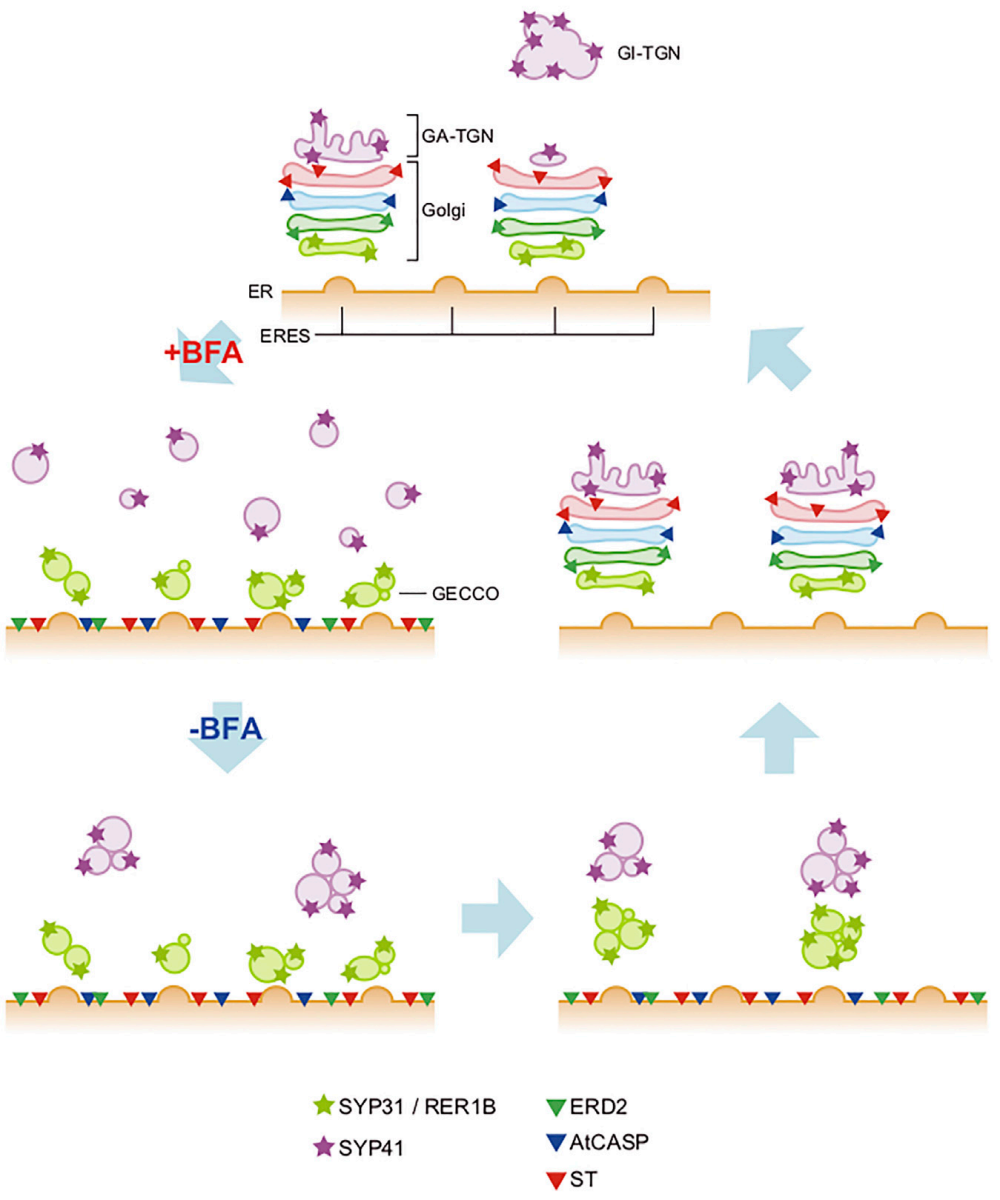
Behaviors of Golgi and TGN upon BFA treatment and washout in plant cells. In tobacco BY-2 cells, BFA-treatment gives distinct effects to the *cis*-most Golgi, the Golgi main body, and TGN (+BFA). The main-body Golgi (with the resident proteins shown by triangles) is completely absorbed into the ER, whereas *cis*-most Golgi, marked by SYP31 and RER1B (light green stars), remains as punctate structures near the ERES, which we designate GECCO (Golgi entry core compartment). TGN (marked by purple stars; SYP41), on the other hand, gets fragmented into small vesicles and dispersed in the cytoplasm. When BFA is washed out (-BFA), these components regenerate the original structures but their processes are again quite different. The Golgi main body reforms stacks in the *cis*-to-*trans* direction using the GECCO as the regeneration scaffold. TGN reassembles by itself independent of the GECCO and the Golgi main body, but finally meets Golgi and starts staying side by side (Ito et al., 2012; Ito et al., 2017; Ito et al., 2018b). GA-TGN, Golgi-associated TGN; GI-TGN, Golgi-independent TGN (see Figure 3).

Similar effects of BFA on the Golgi have been seen in plant cells as well (Ritzenthaler et al., 2002; Langhans et al., 2007); that is, most of the Golgi components are relocated to the ER. However, Ito et al. (2012) discovered that in tobacco BY-2 cells some *cis*-components such as SYP31 (plant counterpart of yeast Sed5 and animal Syntaxin-5) and RER1 (Golgi-to-ER retrieval receptor) remain associated with small punctate structures on BFA treatment, which act as the scaffold of Golgi regeneration when BFA is washed out (its action is known to be reversible) (Figure 2). Since components of the main Golgi body traverse this specialized *cis*-most compartment during Golgi regeneration, the name GECCO, standing for Golgi entry core compartment, was proposed for this compartment (Ito et al., 2018b; Ito and Boutté, 2020). The role of GECCO can be regarded similar to that of ERGIC. In fact, in mammals, ERGIC is known to remain unabsorbed to the ER upon BFA treatment (Lippincott-Schwartz et al., 1990; Saraste and Svensson, 1991; Tang et al., 1993; Füllekrug et al., 1997). In the case of plant cells, the Golgi is almost always located in the vicinity of ERES (daSilva et al., 2004; Sparkes et al., 2009; Takagi et al., 2020) and thus a long-way connection is not necessary (Robinson et al., 2015). The existence of such similar compartments at the interface between the ER (ERES) and the Golgi (*cis*-Golgi) suggests the conservation of fundamental architecture.

Recently, Fujii et al. (2020b) showed in Drosophila cells that knockdown of Arf GEF Garz also induces dynamic relocalization of Golgi proteins to the ER, while leaving behind GM130, a component of *cis*-Golgi, in the cytoplasm, again suggesting the existence of a GECCO-like compartment in invertebrate cells (see Figure 4C). It should be mentioned here that mammalian GM130 was also shown to stay in BFA-resistant structures together with ERGIC (Nakamura et al., 1995).

What about the budding yeast then? As the yeast *S. cerevisiae* does not stack its Golgi cisternae, it is not easy to describe the behavior of the Golgi as a whole, but we noticed that Sed5, one of the *cis*-most Golgi components, showed repeated approach and contact to the ERES (Kurokawa et al., 2014). During this action, which we named hug and kiss, cargo is handed over from the ERES to the *cis*-Golgi. This finding also suggests that the *cis*-most compartment of the yeast Golgi has a special role for the delivery of cargo from the ERES, like GECCO in plant cells and ERGIC in animal cells.

The effects of BFA may be also informative to define such a compartment. Indeed, our preliminary experiments on yeast indicate that there is a group of *cis*-Golgi proteins that do not relocalize to the ER by BFA (Tojima and Nakano, unpublished). These proteins would be good candidates to constitute the yeast counterpart of GECCO.

On the ERES side, COPII proteins are considered to play roles in the selection of cargo (Aridor et al., 2001; Sato and Nakano, 2005; Miller and Barlowe, 2010). As activation of Sar1 GTPase is essential for the COPII assembly, Sar1 was long thought to be present all over the COPII lattice (Miller and Barlowe, 2010; Gillon et al., 2012). However, recent *in vivo* and *in vitro* studies (Kurokawa et al., 2016; Iwasaki et al., 2017) have shown that Sar1 is mostly excluded from the completed COPII cage and present only at the rim or edge regions. This suggests that the uncoating of COPII could take place independently of the vesicle release from the ER (Raote and Malhotra, 2019).

Recent studies also indicate that the coated cage of COPII is very flexible (Mironov et al., 2003; Palmer and Stephens, 2004; Gillon et al., 2012; Raote et al., 2021; Shomron et al., 2021) and can accommodate very large cargo such as collagen or chylomicron in mammalian cells (Raote and Malhotra, 2021). The COPII buds may even be capable of conveying cargo to the next compartments (ERGIC/GECCO and further to the Golgi) before they physically detach from the ER (Raote and Malhotra, 2019). The wonderful morphological work on HeLa cells by Weigel et al. (2021) demonstrated amazing structures extending from the ERES, which indicated VTC-like profiles on the top of ERES. Pearled tubular membrane intermediates are occasionally seen, which probably connect to the Golgi, suggesting a very dynamic nature of the ERGIC compartment.

While admitting the importance of the long-distance transport from the peripheral ERES to the perinuclear Golgi ribbon, we can observe even in mammalian cells that ERES and ERGIC are very abundant in the vicinity of the Golgi ribbon. The cargo trafficking in mammals may actually be performed largely in the pericentrosomal region where the ER and the Golgi are very close to each other. We should not underestimate the short-distance processes (Saraste and Marie, 2018).

An intriguing question is whether GECCO/ERGIC is a part of the Golgi apparatus or an independent organelle (see Marie et al., 2012; Ito and Boutté, 2020). Considering the common role of this compartment, I suppose that the position in very close vicinity to both the ER and the Golgi is the classic, default pattern, that is conserved during evolution. The fact that the ministacks of mammalian Golgi produced by depolymerization of microtubules become located in front of ERES (Cole et al., 1996) supports this idea.

### The Main Body of the Golgi

Here I am not going to discuss the mechanism of cargo transport within the Golgi. It is widely accepted now that cisternal maturation is the major mechanism for anterograde movement of cargo (Glick and Nakano, 2009; Nakano and Luini, 2009). The roles of COPI and tubular connections (Yang et al., 2011; Ishii et al., 2016) have been shown, but disputes remain. Their clarification will need more critical experiments. Recent yeast studies by precise live imaging revealed that secretory cargo sometimes shows backward movement, suggesting cargo recycling in or around the Golgi (Casler et al., 2019; Kurokawa et al., 2019). To understand the underlying mechanisms, it is probably necessary to consider the roles of TGN.

Enzymes involved in glycosylation/modification reactions are considered as regular members of the main body of the Golgi. It is probably safe to postulate that sequential reactions go on when cargo proteins proceed across local regions from cisterna to cisterna. However, there are multiple glycosylation reactions that could occur in parallel, for example, *N*-linked and *O*-linked modifications. Whether such different series of reactions take different paths in the Golgi is another exciting question to be pursued (Yano et al., 2005; D’Angelo et al., 2013).

Besides glycosylation enzymes, some non-enzyme components have been mapped in the *trans* region of the Golgi. They include lipid-transferring proteins such as OSBP (oxysterol-binding protein), CERT (ceramide transfer protein) etc. (Levine and Munro, 2002; Hanada et al., 2007), and perhaps some Arl and Rab GTPases. As these protein functions could be executed in TGN as well, careful examination may be necessary when discussing their exact localizations.

### *Trans*-Golgi Network

The name *trans*-Golgi network was given to the morphologically prominent reticular structure found at the *trans* side of the Golgi stack (Roth et al., 1985; Griffiths and Simons, 1986; Taatjes and Roth, 1986; Geuze and Morré, 1991), which was regarded as a part of the Golgi for some time. A variety of cargo carriers including clathrin-coated vesicles were found in this area, and the concept that this compartment is important for sorting of cargo for different destinations was established (Griffiths et al., 1989; Keller and Simons, 1997; Hinners and Tooze, 2003; Bard and Malhotra, 2006; De Matteis and Luini, 2008; Gonzalez and Rodriguez-Boulan, 2009; Chen et al., 2017).

**FIGURE 3:**
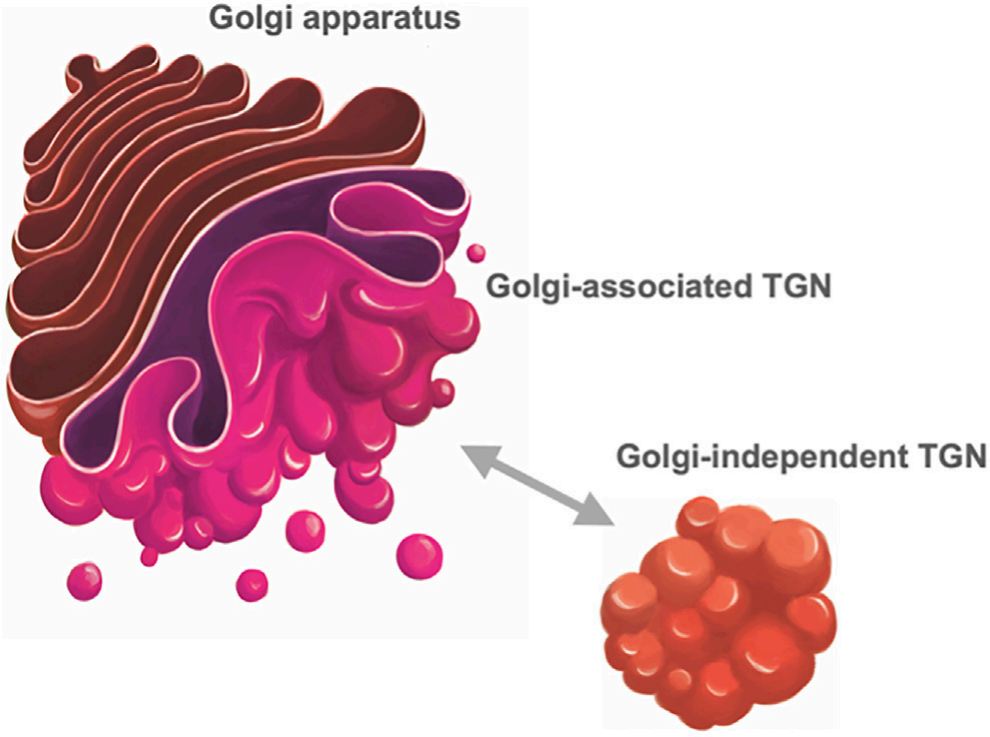
*Trans*-Golgi network of plant cells. In plant cells, TGN (*trans*-Golgi network) takes two statuses, one being attached to the *trans* face of the Golgi (Golgi-associated TGN) and the other detached from the Golgi and free in the cytoplasm (Golgi-independent TGN) (Staehelin and Kang, 2008; Uemura et al., 2014). These two statuses are interchangeable; Golgi-independent TGN can dissociate from the Golgi and reassociate with it.

Now, accumulating evidence tells us that TGN should be considered as an organelle independent of the Golgi. The most striking discovery was that TGN can exist away from the Golgi in plant cells (Uemura et al., 2004; Staehelin and Kang, 2008; Viotti et al., 2010; Uemura and Nakano, 2013). SYP4 and SYP6 subfamilies of plant SNARE proteins (corresponding to Tlg2 and Tlg1 proteins in yeast and Syntaxin-16 and Syntaxin-6 proteins in mammals, respectively) are good markers of TGN. Uemura et al. (2004) realized that when transiently expressed in Arabidopsis protoplasts they mark structures clearly distinct from the Golgi. Such distinction of Golgi/TGN was also seen in living Arabidopsis root tissues, although the extent of displacement depends on the developmental stages of cells (Uemura et al., 2012; Uemura et al., 2014). Furthermore, the live imaging of Arabidopsis TGN demonstrated that the Golgi-independent free TGN can dissociate from the Golgi-associated TGN and repeat reattachment to and dissociation from the Golgi (Uemura et al., 2014) (Figure 3).

Another surprise about the plant TGN was that it acts as a very early compartment of endocytosis. A lipidic dye FM4-64, a popular marker for endocytosis, was shown to reach the TGN much earlier than other compartments (Dettmer et al., 2006; Lam et al., 2007; Chow et al., 2008). FM4-64 was also recognized to label punctate structures in yeast very early after entry (Vida and Emr, 1995; Best et al., 2020), which turned out to correspond to TGN (Day et al., 2018). Receptor-mediated endocytosis was also shown to take the direct route from the plasma membrane to the TGN in yeast (Day et al., 2018; Nagano et al., 2019) and plant cells (Viotti et al., 2010). These findings indicate very important roles of TGN during the early steps of endocytosis, at least for plant and yeast cells.

In mammalian cells, endocytic trafficking involves different routes of recycling, very quickly from the early or sorting endosomes and in a more delayed fashion from the recycling endosomes (RE) (Spang, 2009; Poteryaev et al., 2010; Taguchi, 2013; Scott et al., 2014). I will discuss the close relationship between RE and TGN later.

A group of bacterial toxins are known to take a retrograde trafficking route from the plasma membrane to the Golgi and further to the ER (Sandvig and van Deurs, 1996; Bujny et al., 2007). An early process of this retrograde pathway involves transport from the early endosomes to TGN, which is also utilized for recycling of a variety of cargo receptors. Retromer (and its functional homologs) and GARP (Golgi-associated retrograde protein complex) are important players in this traffic and very well conserved from yeast to plants and animals (Seaman et al., 1998; Sannerud et al., 2003; Bonifacino and Rojas, 2006; Bonifacino, 2014; Schindler et al., 2015; Cullen and Steinberg, 2018; Heucken and Ivanov, 2018; Ishida and Bonifacino, 2019; Ma and Burd, 2020).

All these findings indicate that the role of TGN as the trafficking platform is very important not only for exocytosis/secretion but also for endocytic recycling. To achieve such complicated roles of cargo sorting, TGN must have differentiated domains or zones to perform individual functions.

We have been trying to dissect compartments within TGN in the budding yeast by applying 3D and 4D live imaging approaches, which were powerful in demonstration of cisternal maturation (Kurokawa et al., 2013; Kurokawa and Nakano, 2020). By examining the transition of residents in individual cisternae, we can define the order of events in Golgi and TGN. By the extensive analysis of more than 20 constituents of yeast Golgi and TGN (Tojima et al., 2019; Tojima and Nakano, unpublished), we have proposed to classify yeast TGN proteins into two groups, the early TGN for cargo reception and the late TGN for carrier formation. Tlg2 (the homolog of mammalian Syntaxin-16 and plant SYP41/42/43) is the representative of the early TGN, where a variety of cargo flow in, not only from the Golgi but also from endosomes and the plasma membrane. The late TGN produces carriers with different destinations, marked by clathrin, AP-1, GGA, and exomer, and the Arf GEF Sec7 appears to be mostly overlapping with this area.

In distinguishing TGN from Golgi, BFA is again a good tool. Early studies on mammalian cells showed that BFA caused the absorption of Golgi proteins to the ER but did not affect TGN and endosomes in that way and gave rise to tubular clusters independent of the ER/Golgi hybrid compartment (Chege and Pfeffer, 1990; Lippincott-Schwartz et al., 1991; Wood et al., 1991). In later studies on plants, Ito et al. (2017) showed that BFA treatment of tobacco BY-2 cells caused relocation to the ER of most of proteins of the Golgi main body, leaving GECCO compartment behind. Under the same conditions, TGN proteins such as SYP41 (Tlg2 homolog) were not absorbed to the ER but became dispersed in the cytoplasm as numerous small vesicles, which is not exactly the same but similar to the situation seen in mammalian cells (Figure 2). As the effect of BFA is reversible, regeneration of Golgi and TGN was observed after BFA washout, but their behaviors turned out quite distinct. They gradually reformed Golgi and TGN, but in an independent fashion without significantly overlapping for a certain period of time. After a long time of recovery, they meet again and stay side by side, re-establishing the next-door relationship (Figure 2). Thus, we concluded that the Golgi stack and the TGN have quite different origins and properties but somehow collaborate with each other (Ito et al., 2017).

Regarding the positional properties of Golgi and TGN, plant TGN shows two statuses, one associated with Golgi and the other independent of Golgi (Uemura and Nakano, 2013) as mentioned above, and these two statuses appear to be interchangeable (Uemura et al., 2014) (Figure 3).

**FIGURE 4:**
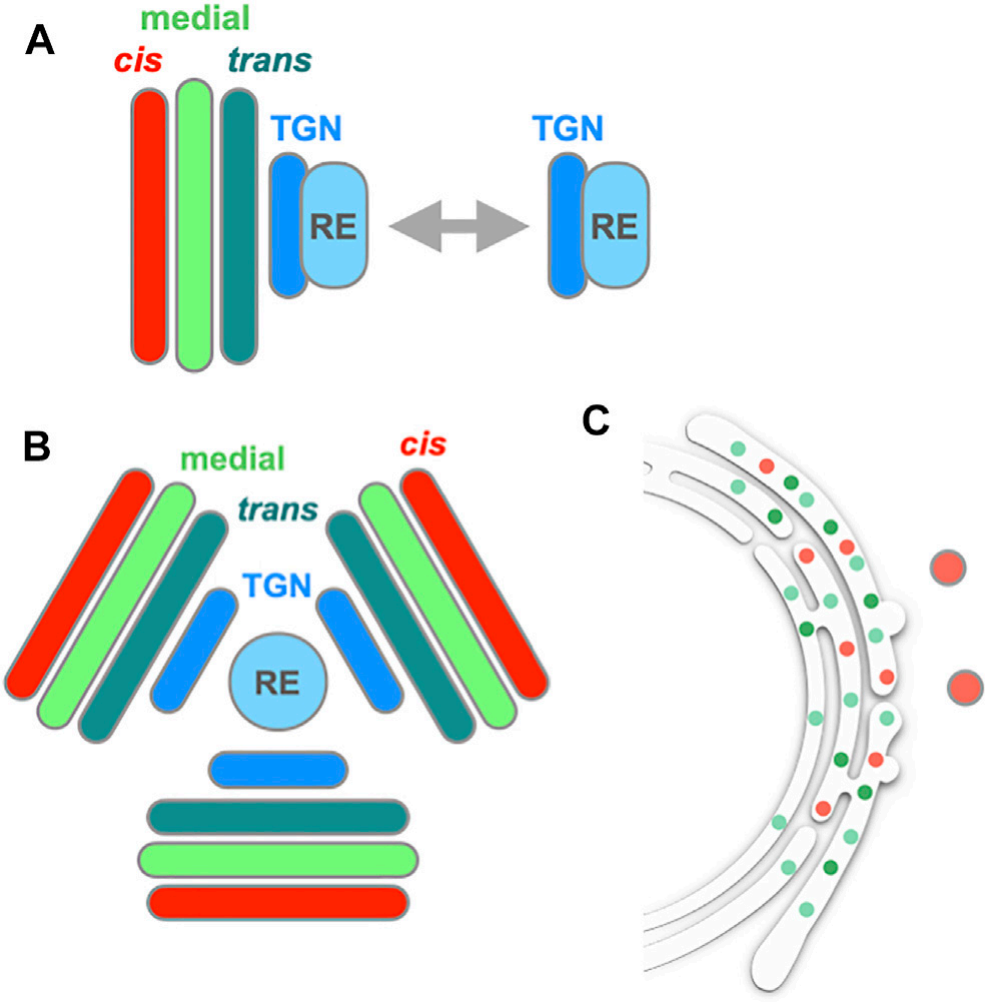
| Behavior of the Golgi, TGN and RE in Drosophila cells. **(A)** In Drosophila S2 cells, Golgi stack (*cis*, medial, and *trans*) and RE (recycling endosome) show attachment and detachment repeatedly (Fujii et al., 2020a). TGN appears to behave together with RE. Similar behavior of RE is also seen in nocodazole-treated HeLa cells. **(B)** When S2 cells are treated with BFA, which inhibits Arf GEF Sec71, Golgi, TGN and RE form a large aggregate (BFA body). In the BFA body, RE sits at the center, TGN follows next and Golgi cisternae align from *trans* to *cis* toward the periphery. **(C)** When the other Arf GEF of Drosophila Garz, which is insensitive to BFA, is knocked down, the Golgi main body is mostly absorbed into the ER, while leaving behind a *cis* component (GM130, red spots) (Fujii et al., 2020b).

Fujii et al. (2020a) recently reported that, in Drosophila S2 cells and nocodazole-treated HeLa cells, Golgi and RE exhibit repeated attachment and detachment (Figure 4A). They called the two states of RE, Golgi-associated RE and free RE. This finding manifests amazing similarity to the case of plant Golgi and TGN. Indeed, markers of TGN and RE show close localization and even partial overlap in Drosophila cells (Fujii et al., 2020a; Fujii et al., 2020b).

BFA treatment of Drosophila cells also gave very interesting results (Fujii et al., 2020b) (Figures 4B,C). Drosophila has two Arf GEFs, called Sec71 (homolog of mammalian BIG and yeast Sec7, which functions in TGN) and Garz (homolog of mammalian GBF1 and yeast Gea1, which functions in *cis*-Golgi). Of the two only Sec71 is sensitive to BFA. When Drosophila S2 cells were treated with BFA, a few gigantic structures emerged, containing Golgi, TGN and RE components (Figure 4B). This kind of BFA-induced large structures are also seen in plant Arabidopsis cells and called BFA bodies (Geldner et al., 2003; Langhans et al., 2011; Nielsen et al., 2012). It should be noted here that the situation is somewhat complicated in plants. Tobacco and Arabidopsis have different sets of Arf GEFs, some of which are BFA-sensitive and others insensitive. GNOM and GNOM-LIKE1 (GNL1) are two major Arf GEFs functioning in and around the Golgi; GNOM is BFA-sensitive in both tobacco and Arabidopsis, while GNL1 is BFA-sensitive in tobacco but insensitive in Arabidopsis (Richter et al., 2007; Teh and Moore, 2007; Robinson et al., 2008; Naramoto et al., 2014). Their sensitivities can be artificially converted by amino acid point mutations (Geldner et al., 2003), but I skip details here to avoid confusion.

Interestingly, detailed examination of such BFA-induced structures in S2 cells reveals that RE is at their center, TGN follows next and Golgi cisternae align from *trans* to *cis* toward their periphery (Figure 4B). Very similar alignment is seen in the BFA bodies of Arabidopsis cells (Langhans et al., 2011). On the other hand, when the other Arf GEF, Garz, is knocked down in Drosophila, most of the Golgi markers are absorbed into the ER (Figure 4C), like the case with mammalian cells (Fujii et al., 2020b). As GNL1, the Arf GEF working in the ER-Golgi traffic in Arabidopsis, is insensitive to BFA, the BFA body formation in Arabidopsis and Drosophila is most likely based on the inhibition of Arf activation only in the post-Golgi processes, leading to the aggregation of the TGN-RE system. The absorption of Golgi into the ER takes place when the Arf activation is inhibited early in the ER-Golgi traffic. These findings imply that the endomembrane system could be classified into two groups, the ER-Golgi system and the TGN-endosome system (see also Lippincott-Schwartz et al., 1991; Manolea et al., 2008; Marie et al., 2009), and the failure to control their organization by compromising Arf activation leads to the jumbling of membranes within each system. If so, there must be a boundary between the main body of Golgi and TGN.

Maturation of compartments appears to go on rather smoothly in the budding yeast and it is not easy to draw a line between the main Golgi body and the TGN. As the order of events we have analyzed relates only to the temporal overlapping and separation of residents, we will probably need to consider spatial segregation of the zones involved in different sorting reactions even in the regions that show up at similar timing.

**FIGURE 5:**
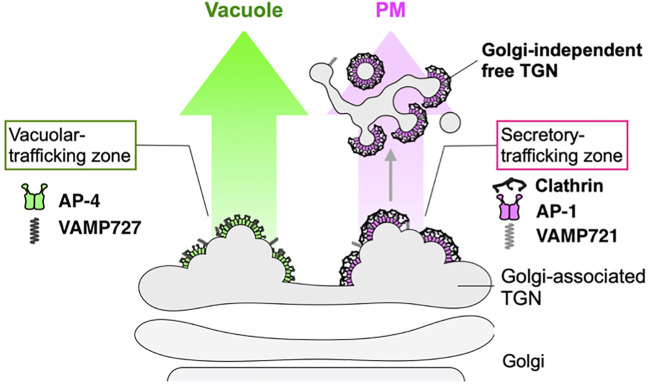
Sorting zones in plant TGN differentiated for secretory and vacuolar trafficking. Cargo sorting events in root epidermal cells of Arabidopsis have been analyzed in detail by state-of-the-art live imaging (SCLIM) (Shimizu et al., 2021). TGN-localized proteins exhibit spatially and temporally distinct distribution. VAMP721 (R-SNARE), AP−1, and clathrin which are involved in secretory trafficking compose an exclusive subregion, whereas VAMP727 (R-SNARE) and AP-4 involved in vacuolar trafficking compose another subregion on the same TGN. These findings indicate that the single TGN has at least two subregions, or “zones”, responsible for distinct cargo sorting: the secretory-trafficking zone destined to the plasma membrane (PM) and the vacuolar-trafficking zone.

At the crossroad of multiple pathways, machineries in TGN are waiting for coming cargo and will then sort them out to different destinations. To gain insights into such differentiation of sorting machineries, the high-speed and super-resolution live imaging technology we have developed (SCLIM) is extremely useful (Kurokawa et al., 2013; Kurokawa and Nakano, 2020; Tojima et al., 2022). We have recently demonstrated that Arabidopsis TGN harbors at least two distinct zones, one containing AP-1 and clathrin for secretory trafficking and the other containing AP-4 for vacuolar trafficking (Shimizu et al., 2021) (Figure 5). This kind of state-of-the-art visualization analysis is expected to unveil dynamic sorting events going on real time in living cells.

### An Attempt to Draw a Simple Common Model

**FIGURE 6:**
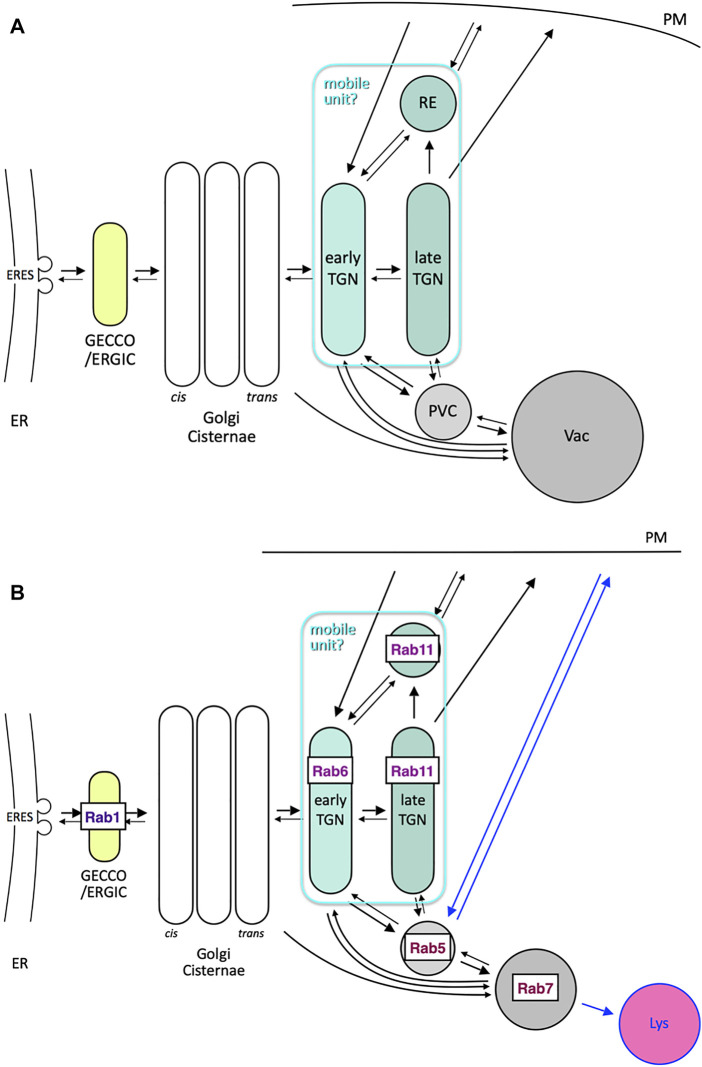
Comprehensive models of trafficking around Golgi and post Golgi. **(A)** Trafficking pathways around Golgi and its next-door neighbors (GECCO/ERGIC and TGN/RE) as well as post-Golgi trafficking to vacuole (Vac) *via* prevacuolar compartment (PVC). Arrows are drawn in an attempt to explain the observations described in this review. Note that early TGN, late TGN, and RE can behave together as a mobile unit, which takes Golgi-associated and Golgi-independent statuses. **(B)** Consideration of conserved Rab GTPases as signboards of compartments, which may be common to yeast, plant, and animal cells. Rab1 (Ypt1 in yeast and RABDs in plants) sits on GECCO/ERGIC, Rab6 (Ypt6 in yeast and RABHs in plants) is probably a good marker of early TGN, and Rab11 (Ypt31/32 in yeast and RABAs in plants) is present in late TGN and RE. The cases of Rab5 (Vps21 in yeast and RABFs in plants) and Rab7 (Ypt7 in yeast and RABGs in plants) are complicated. They mark PVC (MVB) and vacuole, respectively, in yeast and plant cells, but are considered to reside in early and late endosomes, respectively, in mammalian cells (see also Table 1). Blue arrows, i.e., back and forth between plasma membrane and Rab5 compartment and from Rab7 compartment to lysosome (Lys), could be considered inventions in mammals.

As has been discussed above, despite the seemingly big differences in the trafficking architectural systems between animal, plant and yeast cells, there are also significant similarities in many aspects. In Figure 6A, I summarize the routes connecting the Golgi and its next-door neighbors as well as post-Golgi trafficking to vacuole via the prevacuolar compartment (PVC), which can explain most of the observations I have described above. What makes things confusing may be the names of compartments adapted in different systems. A good example is endosomes. Early, late and recycling endosomes do not necessarily refer to the compartments comparable among different species. Let me try to simplify from a different point of view.

Here I propose to consider the localization of major Rab GTPases (Figure 6B). In contrast to Sar/Arf GTPases, which are necessary for vesicle budding, Rab GTPases are generally thought to play roles in targeting and fusion of vesicles (Segev, 2001). In addition, as they collaborate with tethering and fusion machinery, they are often good markers of target compartments (Segev, 2001; Zerial and McBride, 2001).

Let’s pay attention to two Rabs involved in the endocytic pathway, Rab5 and Rab7. These Rab GTPases are extremely well conserved among species. In mammalian cells, Rab5 marks early endosomes and Rab7 is on late endosomes. In yeast and plant cells, however, the situation is quite different. As described above, accumulating evidence indicates that TGN functions as an early endosome. Rab5 is located at PVC and Rab7 is on the vacuole (Saito and Ueda, 2009; Borchers et al., 2021). The transition from the Rab5-to the Rab7-compartment has been well investigated not only in mammals but also in yeast and plants (Rink et al., 2005; Sriram et al., 2006; Balderhaar and Ungermann, 2013; Ebine et al., 2014; Takemoto et al., 2018; Casler and Glick, 2020; Borchers et al., 2021). A big difference between mammals and plants/fungi is the presence of lysosomes (de Duve, 1983; de Duve, 2005). If lysosomes are regarded as an organelle specialized in very efficient degradation in an extremely acidic milieu, the common roles of Rab5 and Rab7 compartments could be conserved in all species.

Mechanisms of intraluminal vesicle (ILV) formation by ESCRT (endosomal sorting complex required for transport) machinery are well understood (Raiborg and Stenmark, 2009; Henne et al., 2011). It gives rise to multivesicular bodies (MVB) or multivesicular endosomes. In mammals, MVB sounds like a synonym of late endosomes (Rab7 compartment), but we should remember that ILV formation begins already in early endosomes (Rab5 compartment). In yeast and plant cells, Rab5 compartment is the MVB (Goh et al., 2007; Haas et al., 2007; Russell et al., 2012). Furthermore, endocytosed cargo is first delivered to TGN and then transferred to the Rab5 compartment, leading to the concept that Rab5 resides in late endosomes in yeast and plants (Cui et al., 2016; Day et al., 2018). Plant Rab5 is further diversified into two types, one directing the route to the vacuole and the other for recycling back to the plasma membrane (Ueda et al., 2001; Ebine et al., 2011; Sakurai et al., 2016; Ito et al., 2018a). Why has the Rab5 compartment changed its role so much during evolution?

In and around the Golgi, several other Rab GTPases are also very important. Rab1 (Ypt1 in yeast) is essential for ER-to-Golgi transport, although it may also be involved in a later process within the Golgi (Segev et al., 1988; Saraste and Goud, 2007; Suda et al., 2013; Gustafson and Fromme, 2017; Suda et al., 2018; Tojima and Nakano, unpublished). The role of Rab6 is somewhat controversial but it is important for receiving cargo in the late Golgi or TGN from endosomes (Liu and Storrie, 2012, 2015; Suda et al., 2013; Thomas et al., 2021). Rab11 is recognized as a good marker of RE (Welz et al., 2014), but its yeast homolog Ypt31/32 is essential for export of secretory cargo from TGN to the plasma membrane (Jedd et al., 1997). Rab11-positive RE is also shown to serve as an intermediate of anterograde Golgi-to-plasma membrane traffic in Drosophila and mammalian cells (Ang et al., 2004; Satoh et al., 2005; Taguchi, 2013). In plant cells, the Rab11 group has been diversified remarkably. Arabidopsis has 26 Rab11 members out of 57 Rab proteins (Rutherford and Moore, 2002; Nielsen et al., 2008; Saito and Ueda, 2009). This suggests that the Rab11 group can fulfill a variety of functions depending on situations (Feraru et al., 2012; Asaoka et al., 2013; Choi et al., 2013), but the conservation of this group also supports its very fundamental role.

Considering the role of Rab11 in sorting cargo into the secretory pathway, its location best fits with the late TGN we defined in yeast. Rab6, on the other hand, is probably working in the early TGN or at the boundary between Golgi and TGN. The relationships of compartments linked to specific Rab GTPases are summarized in Table 1.

**TABLE 1:** Membrane trafficking compartments and well-conserved Rab GTPases.

System	Standard name	Proposed other names	Rab	Coat assembled	Qa−SNARE
ER-Golgi	ER				Ufe1/Syn18/SYP8
	ERES	tER		COPII	
	ERGIC	IC, GECCO, VTC	Rab1	COPII, COPI	Sed5/Syn5/SYP3
	Golgi				
	*Cis*		Rab1	COPI	Sed5/Syn5/SYP3
	*Trans*			COPI	
TGN-endosome	TGN	ERC			
	Early TGN		Rab6		Tlg2/Syn16/SYP4
	Late TGN		Rab11	GGA, AP, clathrin	
	RE	ERC	Rab11	AP, clathrin	
	EE	SE	Rab5 (A)		Syn12?
	LE	MVB, PVC	Rab5 (F/P)		Pep12/SYP21
			Rab7 (A)		Syn7?, Syn12?
	Vacuole		Rab7 (F/P)		Vam3/SYP22
lysosome	Lysosome				?

The next-door neighbors of the Golgi discussed in this review are marked in green (ERGIC/GECCO) and yellow (TGN/RE). Confusing names, early endosomes (EE) and late endosomes (LE), may be clarified by regarding them Rab5 (light blue)- and Rab7 (purple)-compartments. Color codes: A, animals; F, fungi; P, plants.

Abbreviations: AP, adaptor protein complex; COP, coat protein complex; EE, early endosome; ER, endoplasmic reticulum; ERC, endosomal recycling compartment; ERGIC, ER-Golgi intermediate compartment; GECCO, Golgi entry core compartment; IC, intermediate compartment; LE, late endosome; MVB, multivesicular body; PVC, prevacuolar compartment; RE, recycling endosome; SE, sorting endosome; SNARE, soluble N-ethylmaleimide-sensitive factor attachment protein receptor; Syn, syntaxin; SYP, syntaxin of plants; tER, transitional ER; TGN, trans-Golgi network; VTC, vesicular tubular cluster.

From these considerations, I propose a basic principle of Rab-directed compartmentalization as shown in Figure 6B. Rab1, Rab6, Rab11, Rab5, and Rab7 GTPases are selected as the major Rab members very well conserved in evolution. I would like to place Rab6 and Rab11 in early and late TGN, respectively, but the Rab11 compartment could also be called RE. As described above, the behaviors of late TGN and RE are often very similar (Ang et al., 2004; Taguchi, 2013; Bonifacino, 2014). They might be regarded as different sub-compartments or zones of a kind of continuous large platform. The fact that retrograde trafficking from the Rab5 compartment involves tether complexes GARP and EARP (endosome-associated recycling protein complex) for TGN and RE, respectively, which are slightly different in their subunit composition, may support this idea (Schindler et al., 2015). Since both TGN and RE show very dynamic movement back and forth to the Golgi in animal and plant cells, they can be regarded as a large-sized mobile unit as a whole.

Now, a big difference remains regarding the role of Rab5 compartment. Plenty of evidence exists in mammalian cells showing that this is the earliest compartment where newly endocytosed cargo comes in (Chavrier, et al., 1990; Spang, 2009; Scott et al., 2014). In plant and yeast cells, it operates much later in the endocytic pathway (Cui et al., 2016; Day et al., 2018). On the other hand, there is a significant similarity that retrograde trafficking involving retromer and GARP/EARP takes place from the Rab5 compartment to TGN/RE (Bonifacino and Rojas, 2006; Cullen and Steinberg, 2018; Heucken and Ivanov, 2018; Ishida and Bonifacino, 2019; Ma and Burd, 2020). Should this traffic to TGN/RE be considered as that from early endosomes or from late endosomes?

One explanation may be that mammalian cells have somehow evolved to bypass the TGN/RE route to reach the Rab5 compartment. If so, the classic TGN/RE route for endocytosis might also remain. The presence of the mammalian Rab5 compartment as the early endosome has been so prominent; therefore, other possibilities could have been overlooked. Careful revisiting of old data may uncover new findings. To achieve rapid recycling from the early endosome (Rab5 compartment), mammalian cells utilize Rab4, which is not conserved in plant and yeast cells (Sönnichsen et al., 2000; de Renzis et al., 2002; Franke et al., 2019). The need for rapid endocytosis/recycling could be a driving force to take a shortcut and evade the conventional TGN/RE route. Detailed examination of endocytic processes in other model animals would also be interesting.

## Glossary

AP: adaptor protein complex
BFA: brefeldin A
CERT: ceramide transfer protein
COP: coat protein complex
EARP: endosome-associated recycling protein complex
EE: early endosome
EM: electron microscopy
ER: endoplasmic reticulum
ERC: endocytic recycling compartment
ERES: ER exit site
ERGIC: ER-Golgi intermediate compartment
ESCRT: endosomal sorting complex required for transport
GARP: Golgi-associated retrograde protein complex
GECCO: Golgi entry core compartment
GEF: guanine-nucleotide exchange factor
GGA: Golgi-localized, γ-ear-containing, Arf-binding family of protein
IC: intermediate compartment
ILV: intraluminal vesicle
LE: late endosome
MVB: multivesicular body
OSBP: oxysterol-binding protein
PM: plasma membrane
PVC: prevacuolar compartment
RE: recycling endosome
SCLIM: super-resolution confocal live imaging microscopy
SE: sorting endosome
SNARE: soluble N-ethylmaleimide-sensitive factor attachment protein receptor
Syn: syntaxin
SYP: syntaxin of plants
tER: transitional ER
TGN: trans-Golgi network
VTC: vesicular tubular cluster.
